# The Role of Two Factors of Negative Symptoms and Cognition on Social Functioning in Male Patients with Schizophrenia: A Mediator Model

**DOI:** 10.3390/brainsci13020187

**Published:** 2023-01-23

**Authors:** Zixu Wang, Yuru Ling, Yu Wang, Tingting Zhu, Ju Gao, Xiaowei Tang, Miao Yu, Chao Zhou, Yanmin Xu, Xiaobin Zhang, Xiangrong Zhang, Xinyu Fang

**Affiliations:** 1 Department of Geriatric Psychiatry, The Affiliated Brain Hospital of Nanjing Medical University, Nanjing 210029, China; 2 Suzhou Guangji Hospital, Medical College of Soochow University, Suzhou 215008, China; 3 Affiliated WuTaiShan Hospital of Medical College of Yangzhou University, Yangzhou 225003, China; 4 Department of Neurology, The Affiliated Brain Hospital of Nanjing Medical University, Nanjing 210029, China; 5 Department of Psychiatry, Wuhan Mental Health Center, Wuhan 430012, China; 6 Department of Psychiatry, The Affiliated Xuzhou Oriental Hospital of Xuzhou Medical University, Xuzhou 221004, China

**Keywords:** schizophrenia, deficit syndrome, cognition, social functioning, negative symptoms, mediator model

## Abstract

Objective: This study aims to compare the cognitive function and social functioning in male patients with deficit syndrome (DS) and non-DS, and to explore whether cognitive function serves as a mediator in the relationship between the two factors of negative symptoms (motivation and pleasure (MAP) and expressivity (EXP) deficits, and social functioning in schizophrenia patients. Methods: One hundred and fifty-six male patients with schizophrenia and 109 age- and education-matched normal controls were enrolled in the current study. The Chinese version of a Schedule for Deficit Syndrome (SDS) was used for DS and non-DS categorization. The Brief Psychiatric Rating Scale (BPRS) and the Brief Negative Symptoms Scale (BNSS) were used to assess psychotic and negative symptoms in patients. The Social-Adaptive Functioning Evaluation (SAFE) was adopted to evaluate patients’ social functioning, and a battery of classical neurocognitive tests was used to assess cognition, including sustained vigilance/attention, cognitive flexibility, ideation fluency, and visuospatial memory. Results: We found that male patients with DS performed worse in all four cognitive domains and social functioning compared to non-DS patients. Both total negative symptoms and its two factors were significantly associated with all four domains of cognition and social functioning in male patients. Interestingly, our results indicate that only cognitive flexibility mediates the relationship between negative symptoms and social functioning in schizophrenia patients, but there were no differences between EXP and MAP negative factors in this model. Conclusion: Our findings suggest that DS patients may represent a unique clinical subgroup of schizophrenia, and the integrated interventions targeting both negative symptoms and cognition, especially cognitive flexibility, may optimally improve functional outcomes in schizophrenia patients.

## 1. Introduction

Schizophrenia is a serious mental illness of unknown etiology, with a lifetime prevalence of 1.0% in the world population [1] and 0.6% in China [2]. The main clinical manifestations of this disorder include positive symptoms, negative symptoms, and cognitive deficits. Antipsychotic medications are still the first line of treatment for schizophrenia, but they have shown limited efficacy in patients, especially with regard to negative symptoms and cognitive impairments [3]. In recent years, cognitive impairments and negative symptoms in patients with schizophrenia have attracted growing attention as a psychological treatment target, and an increasing number of clinical trials have aimed to observe the intervention options and efficacy for this frequently neglected subgroup of patients [4,5]. Both cognitive impairments and negative symptoms are consistently related to poor social functioning outcomes and impose a great economic burden on families and society [6,7]. There is ample evidence that supports some associations between negative symptoms and cognitive function [8], but the interplay between negative and cognitive symptoms and their impact on social functioning are highly complex, and multiple factors, such as the heterogeneity of the patient, should be considered [9].

Although negative symptoms have customarily been considered as a cluster of symptoms within schizophrenia, there is still a great deal of heterogeneity within the negative symptoms of these patients [10,11]. Deficit syndrome (DS) is regarded as a clinically homogeneous subtype in schizophrenia patients that mainly manifests as the presence of primary and enduring negative symptomatology, which are trait-like characteristics in the disease [12,13]. A substantial number of studies have indicated that there are striking differences in demographics and clinical and neurobiological characteristics between schizophrenia patients with and without DS. For instance, DS patients had a higher proportion of male sex and summer births than non-DS patients [14,15]. In addition, the differences in brain structures and functions between schizophrenia patients with and without DS further suggest that deficit schizophrenia is a different clinical subtype of schizophrenia [13,16]. Moreover, several lines of investigation have shown that schizophrenia patients with DS performed poorer overall and in individual cognitive dimensions [17,18], but some other studies did not replicate this. A recent meta-analysis found no difference in speed-based cognition between the two subtypes of schizophrenia patients [19]. Chen et al. also found that patients with DS performed as well as those without DS in the cognition of processing speed and attention [20]. Another relatively large study revealed that patients with DS scored worse only in the cognitive domain of verbal memory compared to non-deficit patients [21]. Since contradictory evidence exists, more research is warranted to explore the association between DS and distinct cognition aspects in patients with schizophrenia and further the general knowledge on this subject.

Clinically, negative symptoms in schizophrenia patients comprise five domains, including anhedonia (a lack of experiencing pleasure), asociality (reduced social interactions and initiative with others due to decreased interest), avolition (difficulty in initiating and maintaining goal-directed activities), alogia (a decrease in the quantity of words spoken) and blunted affect (lack of emotional expression) [22]. To our knowledge, existing studies frequently used the total score to count the severity of negative symptoms in schizophrenia and to explore its relationship with cognition. The structured factor analysis using different negative assessment tools supported the two-factor model of depressive symptoms: the expressivity (EXP) deficits (alogia and blunted effect) and the motivation and pleasure (MAP) deficits (anhedonia, avolition, and asociality) [23,24]. In addition, a recent study tested four competing factor models of negative symptoms using confirmatory factor analysis and revealed that the two-factor model appears to be the best-fit model for the measurement-invariant latent structure of negative symptoms in schizophrenia patients [25]. Existing evidence indicates that the two dimensions of negative symptoms showed clinically meaningful differences in presentations and functional outcomes [26]. Moreover, the MAP or EXP dimensions also had differential patterns of association with cognitive function in schizophrenia patients. As proof, MAP is strikingly correlated with goal-directed action and ability and executive functioning, while EXP is associated with overall cognitive impairments [27,28,29]. In previous studies, the use of negative symptoms as a whole presented inconsistent results, which consistently supports the two-factor model of negative symptoms. Hence, the negative symptoms should be evaluated separately to figure out their effect on cognitive function and social functioning.

Restoring social functioning and integrating well into society are the primary goals of schizophrenia patients, while both negative symptoms and cognitive function seriously affect the social functioning of these patients [30,31]. The existing literature has demonstrated that negative symptoms are among the strongest independent predictors of social functioning, above and beyond cognitive function, both in patients at a clinically high risk of psychosis [32] and stabilized schizophrenia patients [33]. Another Japanese study also indicated that negative symptoms were markedly correlated to social functioning, while better cognitive performance in a specific domain was significantly associated with greater social functioning [34]. Although ample evidence also supports that negative symptoms are strongly correlated with cognitive function in schizophrenia [35,36], there are no studies that examine the complex internal relationship between negative symptoms, cognitive function, and social functioning in schizophrenia patients, or that consider the subgroup of DS patients with schizophrenia and the two factors of negative symptoms.

Therefore, we aimed to examine whether schizophrenia patients with DS have poorer cognitive functions and social functions compared to patients without DS, and to explore whether cognitive function serves as a mediator in negative symptoms and social functioning in patients with schizophrenia. We used the two-factor model of the negative symptoms to further investigate whether this mediator model was distinctive between EXP and MAP negative symptom dimensions. Moreover, since the male gender has been regarded as one of the risk factors for DS and there are significant differences in the clinical and biological characteristics between male and female schizophrenia patients [37,38,39], we only included male patients with schizophrenia in the present study to reduce the interference of confounding factors and to increase the homogeneity of patients. The hypotheses of the present study are as follows: (1) schizophrenia patients with DS would present poorer cognitive function and social functioning compared to those without DS, (2) both negative symptoms and cognitive functions may be associated with social functioning in these patients, (3) the cognitive function would mediate the relationship between negative symptoms and social functioning, and (4) the MAP and EXP would show different traits on this mediator model.

## 2. Materials and Methods

### 2.1. Subjects

A total of 156 male schizophrenia patients in this cross-sectional naturalistic study were recruited from the inpatient department of the psychiatry unit of the Affiliated WuTaiShan Hospital of Medical College of Yangzhou University in Jiangsu Province, during the period between August 2014 and May 2020, and were independently screened by two senior psychiatrists using the Structured Clinical Interview for DSM-IV-TR Axis I Disorders. The inclusion criteria were as follows: (1) meet the diagnosis of schizophrenia based on the DSM-IV criteria; (2) aged 20–65 years, (3) have a primary school education or above; (4) receiving stable doses of oral antipsychotic medication for over one year; and (5) with stable clinical symptoms. The exclusion criteria included the following: (1) have physical comorbidities, such as head trauma and patients diagnosed with mental retardation; (2) have comorbid substance abuse or dependence; (3) received physical therapies such as electroconvulsive therapy in the past. A total of 109 healthy controls (HC) with matching age and education level, with no mental illness history, no first-degree relative with mental illness, and without lifetime substance dependence or abuse were enrolled from the local community through advertising. The informed consent form was signed by all participants after the procedure had been fully explained. We also obtained permission from the Institutional Ethical Committee of Wutaishan Hospital (No. 2013ZDSYLL052.0). All procedures were conducted in strict accordance with the Declaration of Helsinki.

### 2.2. Subtypes of DS and NDS Patients

The Chinese version of the Schedule for Deficit Syndrome (SDS) was employed for DS and NDS categorization, which is widely used for identifying deficit syndrome in schizophrenia [40,41]. The Criteria for DS are met by the presence of 2 or more of the following symptoms in SDS: curbing of interests, diminished emotional range, diminished sense of purpose, restricted affect, diminished social drive, and poverty of speech. Moreover, the symptoms must have persisted for 12 months and were not caused by other conditions, including substance abuse, depression, environmental deprivation or side effects of drugs. We excluded patients that had comorbid substance abuse or dependence (through medical records and patient and family members) in the present study. The environmental deprivation and side effects of drugs were assessed based on clinical experience by the senior psychiatrists. The DS patients all had no significant changes in their living environment in the six months prior to hospitalization, and had no significant side effects of drugs such as sedation and Parkinson-like symptoms. In addition, we used the ninth item (depressive mood) in the Brief Psychiatric Rating Scale (BPRS) to exclude patients with significant depressive symptoms.

### 2.3. Clinical Assessments

The psychotic symptoms of schizophrenia patients were evaluated by BPRS, which consists of 18 items on a 7-point scale, ranging from 1 to 7 (from absent to extremely severe). The total score reflects the severity of the psychotic symptoms. The BPRS were divided into four subscales: positive, negative, disorganized, and effect syndromes according to the results of the most comprehensive factor analysis of this scale [13].

The negative symptoms and its two factors were assessed by the Brief Negative Symptoms Scale (BNSS). The BNSS contains 13 items organized into six subscales, including asociality, alogia, anhedonia, blunted affect, avolition, and a control subscale named distress. Each item is rated on a 7-point scale (0 = absent, 6 = severe), while the distress item is scored in the opposite way. According to the two-factor structure of negative symptoms, the total scores on the subscales of asociality, avolition, and anhedonia were calculated as MAP deficit, and the total scores on the subscales alogia and blunted effect counted as EXP deficit [42].

A series of classical neurocognitive tests, including the Trail Making Test-A, B (TMT-A,B) [43], the Wechsler Adult Intelligence Scale—Chinese Revision (WAIS-RC) [44], the Animal Naming Test (ANT) [45], the Stroop Color and Word Test (SCWT) [46], the Controlled Oral Word Association Test (COWAT) [47], the Spatial Processing Test [48], and the Digit Vigilance Test (DVT) [49], were used for cognition assessment in all participants. The Z-scores of each test were used for comparisons between groups [50]. When the raw scores of any of the above tests were inconsistent with the observed behavioral performance on the individual tests, such as TMT-A and TMT-B, the reciprocal of the test value was selected during the transformation of the Z-scores to ensure uniformity of the results in the subsequent statistical analyses. Finally, four cognitive domains were analyzed in the present study, including ideation fluency (COWAT and ANT), visuospatial memory (Spatial Processing Test and WAIS-RC), sustained vigilance/attention (Stroop words only and colors only, TMT-A, and DVT), and cognitive flexibility (Stroop color/word interference test and TMT-B and TMT-B). We selected these cognitive tests and grouped them into four cognitive domains based on previous reports regarding cognitive process assessed by each of the tasks [51,52].

The Social-Adaptive Functioning Evaluation (SAFE) was adopted to evaluate the patients’ social functioning. The SAFE contained 17 items rating scale assessing social adjustment and competence, cooperativeness, impulse control, self-care, and life-skill functioning. The items in the SAFE are rated on a 5-point scale (0 = no impairment and 4 = extreme impairment). The final score was determined by observation, caregiver interviews, and patient interactions, and the higher scores indicate more severe social functioning impairments. This scale is specifically designed for chronic psychiatric patients in an institutional setting [53].

### 2.4. Statistical Analysis

The data analysis in the present study was conducted using SPSS 23.0 software. First, we used the Shapiro–Wilk test to check the normality of the data distribution. Then, we compared the socio-demographic and clinical characteristics among DS and non-DS patients and HC groups using Student’s *t*-test, one-way ANOVA, chi-squared, the Kruskal–Wallis tests or Mann–Whitney U tests as appropriate. Third, Spearman’s correlation analysis was used to determine the associations among negative symptoms, cognitive function, and social functioning. The Bonferroni-adjusted significance tests were used for multiple corrections. Finally, the mediating effect model was built using PROCESS in SPSS to test the hypotheses that cognitive function may have different mediating effects of two factors of negative symptoms on social functioning in these patients. A standard procedure was followed using bootstrap sampling 5000 times, which produced 95% bias-corrected confidence intervals. For the above analysis, the significance level of the *p*-value was set at 0.05 (two-tailed).

## 3. Results

### 3.1. Comparisons among DS and Non-DS Patients and HC Groups

**Figure 1:**
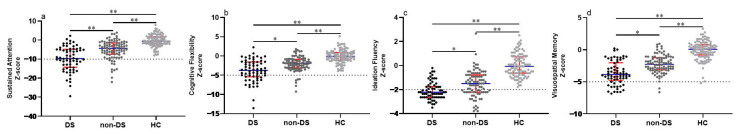
Comparison of cognitive function among DS, non-DS patients, and HCs. (**a**) Comparison of sustained attention among groups. (**b**) Comparison of cognitive flexibility among groups. (**c**) Comparison of ideation fluency among groups. (**d**) Comparison of visuospatial memory among groups. Each bar represents the Median score of cognitive domains. Error bars represent the interquartile range (IQR). Abbreviations: DS = deficit syndrome; HC, healthy controls. * Bonferroni corrected *p* < 0.01 between groups. ** Bonferroni corrected *p* < 0.001 between groups.

Of these 156 male patients with schizophrenia, 66 patients were enrolled in the DS subgroup and 90 in the non-DS subgroup. Table 1 shows the clinical and demographic data among DS, non-DS patients, and HC groups. We found no significant differences in age, education level, and body mass index (BMI) among these three groups (all *p* > 0.05). There was a significant difference in the Chlorpromazine equivalent (Z = 1.992, *p* = 0.046), but no statistical difference in the disease course (*p* > 0.05) between DS and non-DS patients. Compared to non-DS patients, our results reveal that male patients with DS exhibited more severe negative symptoms (negative subscale in BPRS: Z = 10.432, *p* < 0.001; BNSS total score: Z = 9.361, *p* < 0.001; EXP in BNSS: Z = 8.315, *p* < 0.001; MAP in BNSS: Z = 9.451, *p* < 0.001), but no statistical differences in the positive subscale, disorganized subscale and affective subscale scores in BPRS (all *p* > 0.05). We also found that schizophrenia patients had severe cognitive deficits in all four domains, with the most prominent being in the DS patients, followed by the non-DS, when compared to the HC group (See Figure 1). In addition, our results indicate that DS patients had higher SAFE scores compared to non-DS patients (Z = 5.563, *p* < 0.001).

### 3.2. Correlations among Negative Symptoms, Cognitive Function and Social Functioning in Male Patients with Schizophrenia

We conducted the Spearman correlation analysis to examine the relationship among negative symptoms, cognitive function, and social functioning in male schizophrenia patients. The results show that the total BNSS score was positively correlated with the SAFE score (r = 0.404, P_Bonferroni_ < 0.01) and negatively correlated with the score of sustained vigilance/attention (r = −0.426, P_Bonferroni_ < 0.01), visuospatial memory (r = −0.356, P_Bonferroni_ < 0.01), ideation fluency (r = −0.301, P_Bonferroni_ < 0.01), and cognitive flexibility (r = −0.290, P_Bonferroni_ < 0.01) in schizophrenia patients. Furthermore, we found that the SAFE score was negatively correlated with the score of the sustained vigilance/attention (r = −0.328, P_Bonferroni_ < 0.01), cognitive flexibility (r = −0.293, P_Bonferroni_ < 0.01) and visuospatial memory (r = −0.228, P_Bonferroni_ *p* < 0.05). However, there was no significant correlation between the SAFE and ideation fluency in male schizophrenia patients (*p* > 0.05). To further decide whether the two factors in SDS have different correlations with cognitive function and social functioning, we replaced the SDS scores with EXP and MAP scores and performed the above correlation analysis. Our results show that both EXP and MAP were positively related to the SAFE (EXP: r = 0.403, P_Bonferroni_ < 0.01; MAP: r = 0.381, P_Bonferroni_ < 0.01) and negatively correlated with sustained vigilance/attention (EXP: r = −0.363, P_Bonferroni_ < 0.01; MAP: r = −0.433, P_Bonferroni_ < 0.01), cognitive flexibility (EXP: r = −0.247, P_Bonferroni_ < 0.05; MAP: r = −0.299, P_Bonferroni_ < 0.01), ideation fluency (EXP: r = −0.241, P_Bonferroni_ < 0.05; MAP: r = −0.318, P_Bonferroni_ < 0.01), and visuospatial memory (EXP: r = −0.355, P_Bonferroni_ < 0.01; MAP: r = −0.356, P_Bonferroni_ < 0.01).

### 3.3. Mediator Model

**Figure 2:**
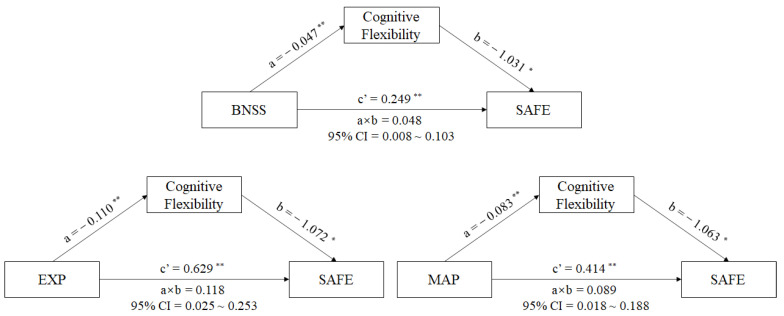
Mediating effects of cognition on the relationship between negative symptoms and social functioning in schizophrenia patients. Note: Path a is independent variable (X) → mediator (M). Path b is mediator (M) → SAFE score (Y), adjusted for X. Path c’ is X → SAFE score (Y), adjusted for M. Path a × b is X→ Y through M. * *p* < 0.01. ** *p* < 0.001. Abbreviations: BNSS = The Brief Negative Symptoms Scale, MAP = the motivation and pleasure deficit, EXP = the expressivity deficit, SAFE = Social Adaptive Functioning Evaluation.

A mediator model with sustained vigilance/attention, cognitive flexibility, ideation fluency, and visuospatial memory being the mediators between negative symptoms (BNSS total score) and the SAFE score in male schizophrenia patients was produced using PROCESS analysis. The results reveal that the standardized total effect of negative symptoms on social functioning was 0.298 (95% CI [0.188,0.407], *p* < 0.001), with the direct effects of negative symptoms on social functioning being 0.249 (95% CI [0.137,0.362], *p* = 0.001), and the indirect effects being 0.048 (95% CI [0.008,0.103]) in the pathway of negative symptoms-cognitive flexibility-social-adaptive ability in schizophrenia patients (See in Figure 2). The interval of indirect effects did not contain 0, which meant that the mediating effect of cognitive flexibility between negative symptoms and social functioning in schizophrenia patients existed. The impairment of cognitive flexibility could aggravate the negative influence of the negative symptoms on social functioning in schizophrenia patients. However, we did not find mediating effects of sustained vigilance/attention, ideation fluency, or visuospatial memory between negative symptoms and social functioning in male schizophrenia patients.

To further understand whether the two factors (EXP and MAP) in BNSS have different relationships in the above mediating model, we replaced BNSS total score with EXP or MAP scores and performed the above mediating analysis. We found that the total effect of EXP on social functioning was 0.746 (95% CI [0.469,1.023], *p* < 0.001), and the direct effect of EXP on social functioning was 0.629 (95% CI [0.346,0.911], *p* < 0.001). There was a striking indirect effect of EXP through cognitive flexibility on social functioning in male patients with schizophrenia (a × b = 0.118, 95% CI = 0.025~0.253) (See in Figure 2). Similarly, other cognitive components showed no mediating effects between EXP and social-adaptive ability in male patients with schizophrenia. 

In the MAP–cognition–social functioning model, our results showed a significant total effect of MAP on the social functioning of 0.502 (95% CI [0.307, 0.698], *p* < 0.001), with the direct effect of MAP on the social functioning being 0.414 (95% CI [0.213, 0.615], *p* = 0.001). There was a striking indirect effect of MAP through cognitive flexibility on the social-adaptive ability in male schizophrenia patients (a × b = 0.089, 95% CI = 0.018~0.188) (See in Figure 2). We did not find any other cognitive components in this mediating model.

## 4. Discussion

To the best of our knowledge, this is the first study to consider deficit subgroups and the two factors of negative symptoms when examining the association between negative symptoms, cognition, and social functioning in male patients with schizophrenia. The main findings of the current study are as follows: (1) DS patients performed worse on all four cognitive domains and social functioning compared to non-DS patients. (2) Both the total negative symptoms and its two factors were significantly associated with all four cognitive functioning domains and social functioning, and social functioning was negatively correlated with sustained vigilance/attention, cognitive flexibility, and visuospatial memory in male patients with schizophrenia. (3) Only cognitive flexibility has a mediating effect on the association of negative symptoms (both EXP and MAP) and social functioning in male patients with schizophrenia. This means that the impairment of cognitive flexibility could aggravate the negative influence of negative symptoms on social functioning in schizophrenia.

In the present study, our results reveal that both DS and non-DS patients had poorer cognitive function, including sustained vigilance/attention, cognitive flexibility, ideation fluency, and visuospatial memory, compared to the control subjects. Our findings are largely in agreement with most of the previous studies, all of which reported that schizophrenia patients had severe and extensive cognitive impairment [38,54]. DS patients suffered from a greater extent of cognitive impairments compared to non-DS patients which have been reported by other researchers. Zhang et al. found that DS patients had global and specific domain deficits of cognition, including language, attention, and immediate and delayed memory compared to non-DS patients [18], and Réthelyi et al. also reported that DS patients performed significantly poorer in multiple cognition domains such as working and verbal memory, attention, ideation fluency, and cognitive flexibility than non-DS patients [52]. However, Cascella et al. observed that DS patients only performed worse in the verbal fluency domain compared to non-DS patients [55], and Sum et al. only found a difference in semantic fluency between those two patient groups [56]. Because of the differences in cognitive assessment tools and heterogeneity of the patients, such as the disease course, the results may vary somewhat across studies. Despite all this, the existing studies all supported the fact that the presence of DS is associated with a deterioration of cognitive deficits in schizophrenia patients. Therefore, DS may represent a subtype of schizophrenia, and more evidence should be provided.

We observed that negative symptoms in all patients were significantly correlated with all four cognitive domains, which was also reported in our past work [51]. Other studies that used different cognitive assessment tools also found correlations between negative symptoms and multiple cognition domains in schizophrenia patients [57,58,59]. Moreover, a recent study indicated that rTMS intervention could relieve negative symptoms and cognitive deficits in chronic schizophrenia patients [4], and a solid body of evidence supports that cognitive remediation, defined by the Cognitive Remediation Experts Workshop (2010), is an effective intervention to improve negative symptoms and cognitive impairments in schizophrenia patients [60,61]. These findings provide evidence for a common pathophysiological mechanism of cognitive impairments and negative symptoms in schizophrenia patients, and thus are worth exploring further. It should be mentioned that there is substantial evidence supporting the difference in association to schizophrenia of the MAP and EXP factors of negative symptoms, such as clinical features [62] and prognosis [63]. A recent study also demonstrated that MAP and EXP factors have different cognitive connections [64]. However, we did not replicate this difference. Since limited studies have separated negative symptoms into MAP and EXP factors when exploring the relationship between cognition and negative symptoms in schizophrenia patients, further investigations are required to reveal their relationship.

As we know, there is substantial evidence supporting the negative symptoms in schizophrenia patients that usually exhibit a remarkable influence on social functioning and functional outcomes [32,34], which is also confirmed in our sample of schizophrenia patients. Although previous studies found that DS patients had a poorer quality of life compared to non-DS patients [65], no study explored the differences in social functioning between schizophrenia patients with and without DS. In the present study, we were not surprised to find that DS patients showed more obvious impairments in social functioning compared to those without DS. Both social functioning and quality of life are important prognostic indicators of schizophrenia; hence, the preliminary findings demonstrate that DS patients had a poorer prognosis compared to non-DS patients, and more attention should be paid to this subtype of schizophrenia not only clinically but also in research work. In the current study, we found that both MAP and EXP factors were significantly associated with social functioning in schizophrenia. Our results highlight the equal importance of the two factors of negative symptoms in influencing social functioning in schizophrenia. However, a recent study using network analysis revealed that the MAP factor accounted for the largest proportion of variance if social functioning in schizophrenia patients [66]. Considering the limited but contradictory results on the relationship between the two factors of negative symptoms and social functioning, more studies are required to investigate this topic in schizophrenia patients.

Cognitive impairment persists throughout the course of schizophrenia, including the premorbid period [54,67]. Existing evidence has indicated that cognitive function, such as executive function and verbal and working memory, could affect social function and quality of life in schizophrenia patients [31,68]. We also found that sustained vigilance/attention, cognitive flexibility, and visuospatial memory were strongly related to social functioning in patients with schizophrenia, thus supporting that cognitive impairment in schizophrenia patients could prevent them from reintegrating into society. Interestingly, a recent study demonstrated that negative symptoms have a much stronger impact on global social functioning compared to the role of cognitive function on social functioning in schizophrenia [33]. Further considering the relationship between cognitive or social functioning and negative symptoms in schizophrenia found in our study, we tested whether cognitive function could play a mediating role in the association between negative symptoms and social functioning in these patients. We did confirm this hypothesis and found that cognitive flexibility, but not other cognitive components, mediates the relationship between negative symptoms and social functioning. Additionally, there were no differences in the mediating effects between the two factors of negative symptoms in the patients. Taken together, the integrated interventions targeting both negative symptoms and cognition, especially cognitive flexibility, may optimally improve functional outcomes in schizophrenia. We know that negative symptoms and cognitive impairments remain the major therapeutic challenge in patients with schizophrenia, and pharmacological treatment options appear to be somehow limited to the negative symptoms and cognitive impairments in these schizophrenia patients. To date, several nonpharmacological interventions have been developed, but with conflicting results. Therefore, the Schizophrenia Section of the European Psychiatric Association (EPA) proposed a guidance paper to provide recommendations for the treatment of cognitive impairment and negative symptoms in schizophrenia patients. Since cognitive remediation and aerobic exercise are both recommended for the treatment of cognitive impairment and negative symptoms in schizophrenia by the EPA [69,70], more studies are required to investigate their further effects on social functioning in these patients. These future studies are encouraged to uncover the common biological mechanisms underlying negative symptoms and cognitive function, refine treatment strategies, allow interventions with greater precision, and promote the recovery of patients with schizophrenia.

The current study had several limitations: First, the cross-sectional design of the study precludes the causal relationship among depressive symptoms, cognitive function, and social functioning in schizophrenia patients. Second, the results may not extend to outpatients since only inpatients were included in the current study. Third, we only recruited male schizophrenia patients, which impaired the generalizability of the findings to the whole population. Whether the preliminary results may be confirmed in female patients is yet to be investigated. Fourth, although a battery of classical neurocognitive tests was used in the present study, they could not include all aspects of cognitive function. Hence, a replication of our findings in more comprehensive tools would be favorable in this regard. Fifth, we did not use dedicated tools for the assessment of depressive symptoms, environmental deprivation, or side effects of drugs in schizophrenia patients to better reduce the interference with the primary negative symptoms. Sixth, we only measured neurocognition and not social cognition performance in the present study, future studies should focus on both neurocognition and social cognition when investigating the present issue.

## 5. Conclusions

In summary, our study observed that male patients with DS had more severe impairment in cognition and social functioning compared to non-DS patients, suggesting that DS patients may represent a unique clinical subgroup of schizophrenia. Additionally, we found that negative symptoms could strongly predict cognitive impairment and social functioning in schizophrenia patients. Interestingly, our preliminary findings provide the novelty of showing that cognitive flexibility has a mediating effect on the relationship between negative symptoms and social functioning in patients. However, the MAP or EXP dimensions had no differential patterns of association with cognitive function and social functioning, or in the mediating model created for these patients. Due to the limitations mentioned above, the findings should be interpreted with caution. Future longitudinal studies should include male and female inpatients and outpatients, using a more comprehensive cognitive assessment tool to verify our preliminary findings. 

## Figures and Tables

**Table 1:** Comparisons of demographic and clinical characteristics among DS, non-DS schizophrenia patients, and HC groups.

	DS Patients (N = 66)	NDS Patients (N = 90)	HC (N = 109)	F/t/Z/X^2^	*p*
Age	50.18 ± 8.38	49.69 ± 7.63	51.29 ± 7.56	1.106	0.332
Education level (year)	8.00 (3.00)	8.00 (3.00)	8.00 (3.00)	1.974	0.373
BMI	24.04 ± 3.65	24.91 ± 3.12	24.09 ± 2.22	2.404	0.092
Total disease course (year)	27.82 ± 7.71	26.37 ± 7.91		1.145	0.254
Chlorpromazine equivalent	500.00 (288.75)	545.00 (155.00)		1.992	0.046
BPRS total score	31.50 (5.00)	27.00 (3.00)		7.980	<0.001
Positive subscale	6.00 (2.00)	6.00 (2.00)		0.493	0.622
Negative subscale	12.00 (2.00)	7.00 (1.00)		10.432	<0.001
Disorganized subscale	6.00 (1.00)	6.00 (1.00)		0.089	0.929
Affective subscale	7.00 (1.00)	7.00 (1.00)		0.523	0.601
BNSS total score	45.00 (20.25)	18.00 (12.00)		9.361	<0.001
MAP subscale	24.00 (10.25)	10.50 (7.25)		9.451	<0.001
EXP subscale	16.00 (8.00)	6.00 (5.00)		8.315	<0.001
Sustained attention	−9.70 (9.45) **^△△^	−4.31 (6.01) ^##^	−0.53 (1.25)	108.764	<0.001
Cognitive flexibility	−3.73 (3.88) *^△△^	−1.89 (2.21) ^##^	−0.07 (0.88)	91.855	<0.001
Ideation fluency	−2.21 (1.90) *^△△^	−1.49 (1.43) ^##^	−0.06 (0.67)	135.281	<0.001
Visuospatial memory	−3.86 (2.72) *^△△^	−2.26 (1.89) ^##^	−0.71 (0.72)	130.400	<0.001
SAFE total score	22.50 (12.50)	16.50 (11.25)		5.563	<0.001

Abbreviations: DS = Deficit syndrome, HC = Healthy controls, BMI = Body mass index, BPRS = The Brief Psychiatric Rating Scale, BNSS = The Brief Negative Symptoms Scale, MAP = The motivation and pleasure deficit, EXP = The expressivity deficit, SAFE = Social Adaptive Functioning Evaluation. Data are presented in Mean ± SD or Median (IQR) according to its normal distribution of not. * Bonferroni corrected *p* < 0.01 DS vs. NDS, ** Bonferroni corrected *p* < 0.001 DS vs. NDS. ^△△^ Bonferroni corrected *p* < 0.001 DS vs. HC. ^##^ Bonferroni corrected *p* < 0.001 NDS vs. HC.

## Data Availability

The data presented in this study are available on request from the corresponding author.
